# Theoretical investigation of malaria prevalence in two Indian cities using the response surface method

**DOI:** 10.1186/1475-2875-10-301

**Published:** 2011-10-14

**Authors:** Sayantani Basu Roy, Ram Rup Sarkar, Somdatta Sinha

**Affiliations:** 1 Centre for Cellular and Molecular Biology (CSIR), Uppal Road, Hyderabad, India

## Abstract

**Background:**

Elucidation of the relationships between malaria incidence and climatic and non-climatic factors in a region is of utmost importance in understanding the causative factors of disease spread and design of control strategies. Very often malaria prevalence data is restricted to short time scales (months to few years). This demands application of rigorous statistical modelling techniques for analysis and prediction. The monthly malaria prevalence data for three to five years from two cities in southern India, situated in two different climatic zones, are studied to capture their dependence on climatic factors.

**Methods:**

The statistical technique of response surface method (RSM) is applied for the first time to study any epidemiological data. A new step-by-step model reduction technique is proposed to refine the initial model obtained from RSM. This provides a simpler structure and gives better fit. This combined approach is applied to two types of epidemiological data (Slide Positivity Rates values and Total Malaria cases), for two cities in India with varying strengths of disease prevalence and environmental conditions.

**Results:**

The study on these data sets reveals that RSM can be used successfully to elucidate the important environmental factors influencing the transmission of the disease by analysing short epidemiological time series. The proposed approach has high predictive ability over relatively long time horizons.

**Conclusions:**

This method promises to provide reliable forecast of malaria incidence across varying environmental conditions, which may help in designing useful control programmes for malaria.

## Background

Malaria is one of the world's major micro-parasitic infections in humans killing nearly two million (mostly children) each year, in addition to more than 500 million people living in the epidemic regions. It has been estimated that almost half the world's population is at a risk of the disease [1,2]. Malaria was declared endemic and a major public health problem in 109 countries in 2008 [2]. It is known to be spreading rapidly in different tropical countries of Central and South America, Asia and Africa, with the sub-Saharan Africa credited for 85-90% of deaths [2-6]. In India, malaria is highly endemic in most regions [5,7]. Despite the introduction of control programs in many parts of the world over the past few decades, the impact of malaria on human population continues to increase. Scientific research has improved our understanding of the host-parasite-vector interactions and their biology. However, factors such as the complexities in the life cycle of the parasite, environmental interactions, evolutionary pressure of drugs and control measures contributing to drug resistance of parasite, and migration of population between endemic and non-endemic areas, continue to contribute to the huge burden of morbidity and mortality accompanying the disease. These also present new challenges to researchers and public health professionals to combat the disease. Relating epidemiological and clinical data, collected by researchers and public health professionals using different methods and analysis tools, to the associated biotic and abiotic factors is an extremely daunting task - particularly in a large and, ecologically as well as demographically diverse country like India.

Epidemiological research on micro-parasitic infections such as malaria, is often based on two separate measures of parasite distribution among hosts (humans, in this case) - the incidence of the disease, and the infection prevalence. The incidence or prevalence measures are primarily based on classifying the population under study with respect to a range of factors such as age, sex, social factors, environmental variability etc. The climatic and environmental factors affect production and survival of the vector (mosquito), and the speed of parasitic life cycle [8-12]. Such studies involve a host of modelling approaches based on mathematical and data-based statistical methods [13]. Data-based statistical modeling, based on the available disease prevalence data for different categorizations, is an integrative approach that uses the past data to predict the future trend. The applicability of such models becomes important while assessing their numerical outputs, estimating parameters, and predicting future values from past observations. Much research has gone in the study of statistical models for malaria to describe the relationship between disease incidence and climatic as well as non-climatic factors [10,14-17]. Several techniques, such as, Logistic regression modeling [18], Poisson regression modeling [19], and Binary logistic regression modeling with fractional polynomial transformations [8] have been used to study the disease transmission process under different environmental factors. A combined mathematical-statistical approach (the Liverpool Malaria model), that uses the dynamic transmission models of the SIRS-type framework and statistical methods for correlating environmental dependencies, was developed recently to successfully forecast the evolution of malaria epidemiology in western Africa [20,21].

Given the fact that data collection is mostly restricted to short time scales (months to few years), in recent studies, researchers have used elaborate time series analysis models to show seasonality pattern in malaria incidence, and the Monte-Carlo Markov Chain methods with Bayesian techniques of a-priori probability assignment, to estimate the risk factors [22-24]. However, there exist some drawbacks, which affect the suitability of these models being fitted into the incidence pattern of the disease. For instance, discontinuity is observed in the time series at high temporal resolution while studying extensive dataset for elucidating climatic role in the transmission of malaria in Africa and Europe [25,26]. Quantitative modeling was applied in Punjab, India but its operation at low temporal resolution renders its wider applicability questionable [27]. The induction of linearity between malaria deaths and temperature via fractional polynomial algorithm in this methodology, too, is not obvious for modeling malaria risk with climatic factors. A recent study [28] attempted to deal with these drawbacks by developing a simple non-linear regression methodology in modeling and forecasting malaria incidence in Chennai city, India. This method introduces successively higher powers of the chosen independent variables and leads to a complex model that does not ensure a trade-off between the number and numerical order of terms and, the goodness of fit of the model. Data-specific models with a large number of linear and nonlinear terms are typical features of these models. Epidemiologists developing models to understand the underlying causes, their relative importance, and relationship with the pattern of the disease have not been able to reach a consensus as to which of the several processes in existence would work the best.

In this paper, the incidence of malaria in two cities in southern India - Chennai and Mangalore - in two different climatic zones, are studied using the Response Surface Method (RSM), which was first introduced to obtain optimum conditions in a chemical investigation [29] and then in various optimization problems such as, structural reliability and biochemical processes [30-32]. It is known that malaria incidence in any region is modulated in response to several environmental factors, and these two cities have considerably different levels of disease severity, population density, rainfalls and vegetation patterns. The RSM models, used for the first time to study any epidemiological data, is applied to develop a general framework to forecast malaria incidences by considering the relative effects of environmental factors without *a priori *assumptions on independent or response variables (discrete or continuous). Two types of epidemiological data for malaria cases are considered - i) a three-year time series data of Slide Positivity Rates (SPR) values of malaria for Chennai, and ii) a five-year time series of Total Malaria cases (TMC) for Mangalore. The RSM models delivered good predictive ability over long time horizons. In addition, a step-by-step model reduction technique is proposed to refine the initial model obtained from RSM, which not only provides a simpler structure but also gives better fit as supported by Akaike's Information Criterion [33]. The study on two data sets with different strengths of malaria incidence, reveals that this method can be used successfully to analyse the behavior of epidemiological time series, and the forecasts obtained from the simpler yet improved models show high predictive ability. Hence, this approach leads to detection of crucial environmental factors influencing the transmission of the disease while offering a coherent and integrated understanding of the disease process in any area.

## Methods

### Data

**Figure 1:**
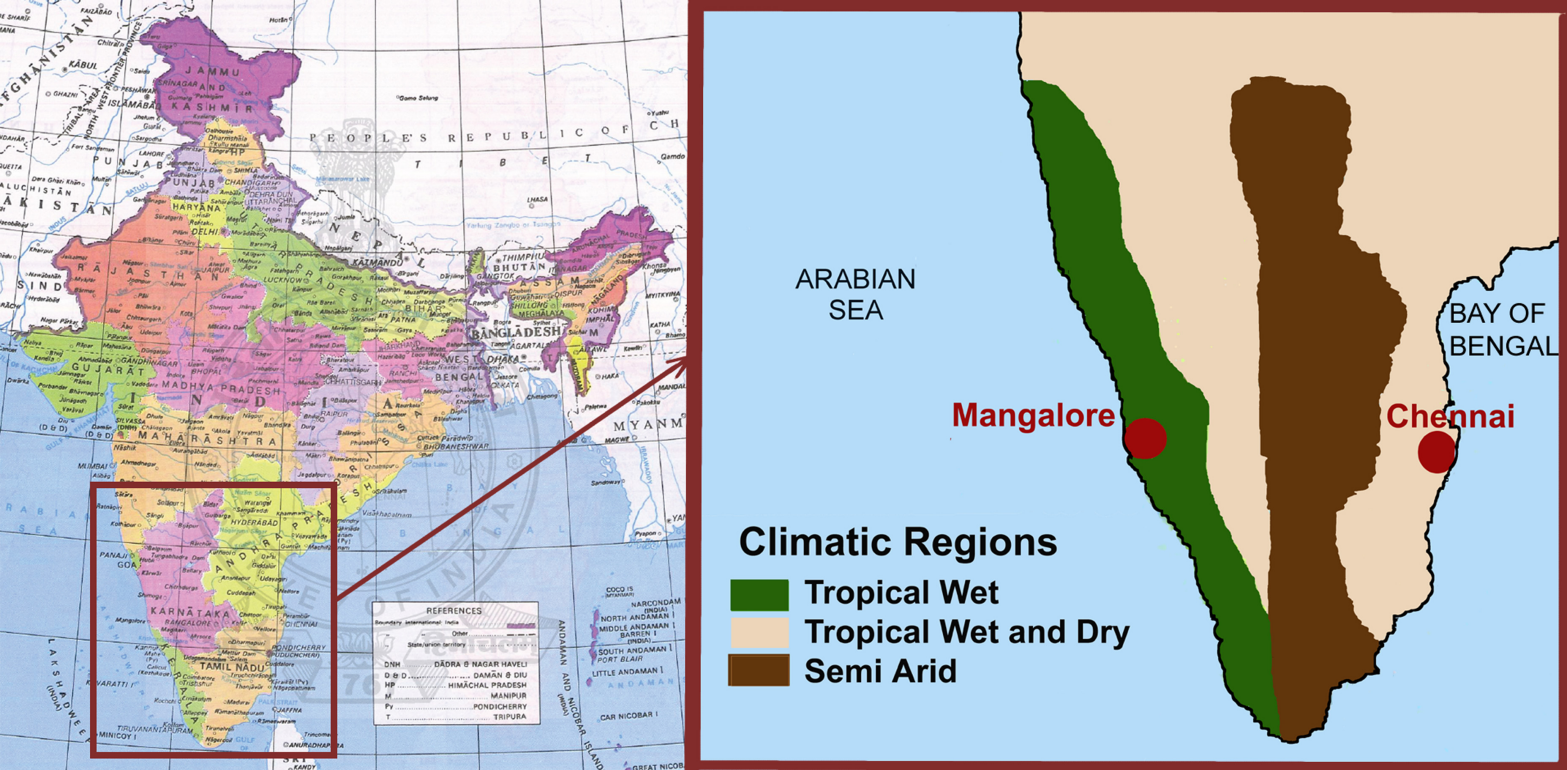
**Geographical location **[34 ]**and climatic regions for Mangalore and Chennai cities**. Map within the box is not to the scale.

Malaria incidence data, along with different demographic and environmental factors, were collected from literature and different other sources for two cities in India - Chennai and Mangalore [28,34-38]. Both cities are situated in southern part of India with Chennai in the coast of the Bay of Bengal and Mangalore near the Arabian Sea (see Figure 1). Even though both the cities have tropical climate, their rainfall pattern is quite different, thereby putting them in two different climate zones - tropical wet for Mangalore and tropical wet-dry climate for Chennai (Figure 1).

Malaria is endemic in both the cities for the last few decades, though Chennai has lower endemicity compared to Mangalore. They are victims of rapid industrialization, witnessing an unprecedented spurt in construction activities in recent years, thus facing the problem of mosquito breeding in man-made clear water sources like wells, overhead tanks, sumps, cisterns as well as other defective and illegal drainage systems. Yet there are several differences between them, specifically in their demographic and climatic factors. Mangalore has less population (398,745) as compared to Chennai (5.6 million, third largest city) according to the 2001 census of India. In addition, the urban area in Mangalore has 32 recognised slums, and nearly 22,000 migrant labours live in slums within the city limits, whereas, Chennai has the fourth largest population of slum dwellers among major cities in India, with about 820,000 people (18.6% of its population) living in slum conditions [35].

Two types of data for malaria incidence, Slide Positivity Rate (SPR) and Total Malaria Cases (TMC), along with different demographic and environmental factors were collected from Chennai and Mangalore. The data for two types of malaria (due to *Plasmodium vivax *and *Plasmodium falciparum*), show similar temporal patterns of disease incidence, and therefore are combined together for further analysis.

#### Chennai city

Slide Positivity Rate (SPR = number of cases found positive/number of blood smears examined) values, the dependent variable under study, were obtained from the Corporation of Chennai, Tamil Nadu, India for two types of malaria, *P. vivax *and *P. falciparum*, for the period January 2002 - December 2004. The temporal scale of SPR is the monthly values collected from the Corporation. The threshold defining the positive slide is calculated based on JSB Stain for thick and thin films, which is standard method used by the laboratories under the National Malaria Eradication Programme in India [37]. A thin blood smear film with parasitaemia value of 2-3% or above is considered to be a positive slide. The number of blood smears collected lies in the range of 16306-63717 depending on the seasons. The number of cases that tested positive was between 1184 and 4275, of the collected smears. Since the number of people that are exposed to the disease is directly proportional to the incidence, the population of the city is considered as an independent variable for this study. Monthly values for population were obtained from a third order polynomial fitting approach described in [28]. The environmental variables, such as, average temperature, average humidity and rainfall values, were also used as other independent variables. Average monthly values for temperature and humidity were taken as the arithmetic mean of minimum and maximum values available. Further details are available in [28].

**Figure 2:**
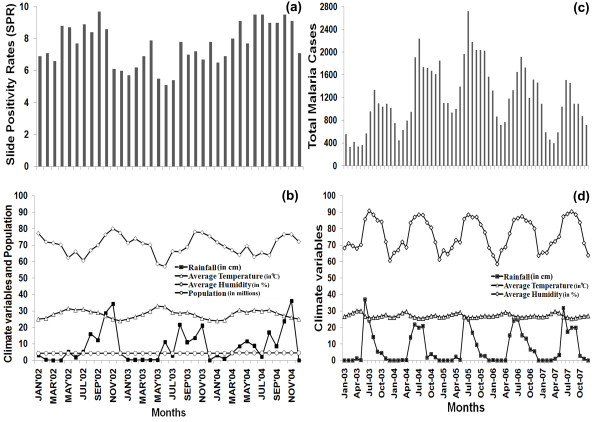
**Malaria incidence and climate data**. **(a) **SPR (%) values in Chennai, during January 2002 - December 2004, and **(c) **TMC values in Mangalore, during January 2003 - December 2007. The climate variables, Rainfall (cm), Temperature (°C), Humidity (%), and Population (in millions) are appropriately scaled to fit in the same plot for: **(b) **Chennai, and **(d) **Mangalore.

The SPR values (bars) and the environmental variables, including population (lines), for Chennai are presented in Figure 2(a and b). Chennai gets most of its seasonal rainfall from the North-East monsoon winds, from mid-October to mid-December, followed by light rainfalls during March to June of the South-West monsoon season showing two clear peaks for rainfall, with late May to early June being the hottest part of the year. This results in two rainfall peaks each year as seen in Figure 2(b). The variation in the levels of the peaks can be attributed to climatic stochasticity. Average humidity variations follow the rainfall pattern and reach a temporary peak just before the start of the first monsoon in May - July, though its variance is low. And, the average temperature oscillates within a fixed range, and increases when the rainfall reaches the high peaks. Population remained relatively invariant during the period of study. Some influence of climatic variations on the malaria incidence pattern can be seen in Figure 2(a), where the SPR values increase with higher rainfall and subsequently decrease during the seasons with sparse rainfall.

#### Mangalore city

Monthly malaria cases from January 2003 through December 2007 (60 time points), were obtained from Mangalore city, Karnataka [37]. These Total Malaria Case (TMC) values (sum of *P. vivax *and *P. falciparum *cases) are the measures of malaria incidence, which are different from the numerical nature of SPR values. The total malaria cases include both symptomatic and asymptomatic cases. Also, average monthly values of temperature, humidity, and rainfall for the same interval were computed from a weather database that gives the daily minimum and maximum values for these variables [38]. Mangalore is under the direct influence of the Arabian Sea branch of the South-West monsoon and has a very high annual precipitation [36]. It receives about 90% of its total annual rainfall between May to September, with occasional rains in October, while remains extremely dry from December to March.

The TMC values and the climate variables are presented respectively as bars and line graphs in Figure 2(c and d). Here, one can observe a more regular pattern of rainfall with a yearly peak (June-August), preceded by a dry season, and followed by gradual decrease. Also, the rainfall pattern is mimicked by humidity, albeit with a smaller variance. Owing to a tropical climate, the temperature shows limited variation. The TMC values clearly follow the rainfall pattern, though there is a consistent lag between the yearly maxima for TMC values and that of rainfall.

It may be noted here that the data for climate variables were obtained from daily weather sources, and they were averaged to monthly scale to ensure compatibility with the monthly parasitological data (SPR and TMC). Being in a tropical climatic region, the climatic variables for both the cities show small daily variations and hence the choice of arithmetic mean for calculating the monthly averages is appropriate and easy to apply. Also, none of the data are measured in logarithmic scale, or are highly skewed, or show high variations, hence geometric mean is not considered here [39].

### Statistical methods

Before the model development is discussed, statistical techniques for exploring the relationships between dependent variables (SPR and TMC values) and independent variables (climatic factors, previous incidence of the disease etc.) are presented. The details are given in Additional File 1 (Sections A-E).

#### Autocorrelation function

Autocorrelation of any time series data describes the correlation and relation in general between values of the series at different points in time [40]. Intuitively for infectious diseases, the autocorrelation *at lag one *is expected to be important among other lags since values at the previous time point tend to correlate closely with those of the current time.

Presence of autocorrelation was studied for the independent variables (SPR and TMC) (Additional File 1, Section A, Figure S1). The value at a particular lag was considered significant if it extends beyond the 95% confidence limits. In addition, Ljung-Box Q statistic (LB) is used to obtain the significance for autocorrelation values [40]. For autocorrelation value, *ρ*_*k *_at lag *k*, for sample size *n *and testing for cumulative autocorrelation for *h *lags, LB is given by

(1) $$Q=n\left(n+2\right)\sum_{k=1}^{h}\left(\rho_{k}^{2}∕\left(n-k\right)\right).$$

Variables with significant cumulative autocorrelations at a particular lag (mostly one) are also considered as independent variables in the model. For SPR and TMC values, autocorrelation values *at lag-one *were found to be high with significant LB values (Additional File 1, Section A, Table S1).

#### Correlation and residual plots

Linear dependencies were quantified using correlation, and *t*-statistic was used for testing of significance. The two-sided *t*-statistic for a correlation, *r *at *α *level of significance is given by

(2) tα2, n−2=r(n−2)(1−r2)

Correlations at different lags (Additional File 1, Section B, Tables S2-S4) were considered to investigate the variation or consistency in the different segments of the data [41]. Further, when correlation was found to be low or non-significant, perhaps indicating non-linearity, Residual Plots were employed for further confirmation. Such plots show non-random distribution of points around the mean axis if the relationship between the variables under consideration is nonlinear. High correlations were observed between SPR and SPR-at-lag-one, and TMC and TMC-at-lag-one. For other combinations of independent and dependent variables, where correlation was found to be low, non-linearity was confirmed by residual plots (Additional File 1, Section B, Figures S2 and S3).

### Model development using Response Surface Method

Response Surface Method (RSM) is a classical optimization technique that integrates mathematical and statistical approaches to the problem of finding the values of several independent variables (*factors*) affecting the optimum response in the variable of interest - the dependent (*response*) variable [42]. This approach has mostly been employed when experiments are conducted by varying the levels of the different factors and observing the effect on the response, training the values in a manner so as to reach the optimum point on the response surface.

The first step in RSM is to find the First Order model with appropriate estimation of model parameters. If this preliminary analysis indicates non-linearity in the relation between independent variables and the response, along with the linear terms, a quadratic polynomial is used to include the second order terms of the independent variables individually and all possible interaction terms of second order so as to propose the Second Order model (see Additional File 1, Section C for details). The method of least squares is then used to obtain an initial estimate of the parameters for the First or Second Order model depending on the cases. The response surface analysis is then performed, using the fitted surface to obtain the values of the parameters that optimize the response value [42]. In this study, this is attained in a complementary manner, by obtaining better, successive forecasts as newer (reliable forecasts) climate variable values are used in the model, thus, optimizing our future estimates of incidences. For obtaining the optimum parameter values following RSM, 80% of the data have been used (28 time points for SPR, 48 time points for TMC), and the remaining 20% of the data are used for validating the model predictions. The hypothesis of using a part of the data for model-building and retaining the rest for validation is common practice in statistical methods and the specific ratio may vary with the size of data at hand, as well as, the nature of data.

The final estimates of the parameters in a model give optimum goodness of fit realized using RSM. Such a model, if Second Order, will have a large number of terms, all of which may not have equal contribution to the variation in response. The presence of more terms also renders the model complicated, and decreases its efficiency statistically as more parameters are estimated from limited data. Hence, there is a requirement for a method to reduce the number of terms in a model without reducing the goodness of fit.

### Model reduction process

A systematic model reduction process is developed to derive a simpler model with fewer but important terms, while ensuring efficiency (coefficient of determination) similar to that obtained in the initial model fitting through RSM. Figure S4 in Additional File 1 (Section C) shows the flow chart of the systematic process followed for model development using RSM and the model reduction technique.

The foremost task in this process is to enlist the resultant orders of magnitude of each term. In a model, each term consists of two components: the variable - linear or quadratic and the corresponding estimated coefficient or parameter value. The resultant order of a term is defined as the sum of the orders of the variable and the parameter in scientific notation. This exercise asserts its importance in cases when there is a great disparity between the orders of the variable and the parameter of a single term. Even though the parameter has very low order, a high order of the variable component may render the resultant order to become higher than that of the response, which may finally lead to its exclusion from consideration for the next step and vice-versa.

Following the above process, the coefficient of determination (R^2^) is examined at each step of the model by removing each term, beginning with the term with the smallest resultant order (Additional File 1, Section C, Tables S5 and S6). A stepwise process is followed, considering removal of terms with successively higher resultant orders, and observing the change in the coefficient of determination (R^2^) at each step, to decide about the retention or removal of the term under consideration. This process is continued till the terms with resultant orders equal to that of the response or higher are reached. Here, the underlying assumption is that the contribution of a term in a particular model equation is proportional to its resultant order. This is similar to backward regression, since the removal of terms from the model is performed once all the independent variables as well as corresponding possible terms up to second order have been included in the model. R^2 ^is employed for the decision of retaining or removing the terms in the model since it is a statistic traditionally used for providing the measure of the proportion of the variation explained by the model [43,44].

The outcome from this method remains unchanged to changes in the unit of the variables. Such a change in a variable is proportionately adjusted in the parameter component of the terms which contain that variable, preserving their resultant orders of magnitude. This may imply re-arrangement of the resultant orders of magnitude of the terms in the model, in turn, leading to a different reduced model. Also, the criterion (that is, the order of decimal place differences in the coefficient of determination considered significant) described above to arrive at the decision of retaining or removal of terms in the model will vary according to the nature of response being studied and variation in the allowed amount of loss in accuracy owing to model reduction.

### Model validation and forecast

To assess the goodness of fit for the initial models using RSM and the reduced models using model reduction technique, four diagnostics tests are carried out: (a) Confidence Intervals for the data points, which are used for fitting the model, (b) Akaike's Information Criterion to determine the better fit model, (c) Prediction Intervals in Forecasting Method for the data points estimated from the reduced model but not used for fitting the model, and (d) Collinearity Analysis for testing homoscedasticity of the residuals.

#### Confidence Intervals

Using Gauss Markov Theorem [41], it is tested whether each estimated value of the data points used for fitting the model (both for the optimized and reduced models) lies within the 95% confidence intervals or not (Additional File 1, Section D).

#### Akaike's Information Criterion (AIC)

This statistic measures the appropriateness of forecasts of the estimated statistical models and selects the better model from the given models [33,40]. The model with the lower (or lowest) AIC is the better (or best) model. For a model based on *n *observations, having *k *parameters and the residual errors denoted as ∈_*i*_,*i = *1,2*,..,n*, the value of the criterion is given by formula (3).

(3) AIC=(−2)max(log(likelihood)+2k

AIC has been used to substantiate the claim that considering R^2 ^for reducing the number of terms in the model does provide us with a model that attains a trade-off between the variation explained and the number of parameters estimated.

#### Prediction Intervals in Forecasting Method

Once the reduced model is obtained, using 80% of the available data, the next step to validate the model is to test the forecasting ability. This is performed by employing the model to predict values for the remaining 20% time points. Since the model incorporates auto-regressive terms (SPR-at-lag-one for SPR values, TMC-at-lag-one for TMC values), this prediction ensures that each estimated response serves as an input for the subsequent estimate. This means that the predictions use the model prediction as the input for successive estimates, in addition to reliable climate forecasts and/or population projections. Further, this feature leads to training the data on hitherto unforeseen terrain, making it more conducive to unknown data. Using ancillary statistics [41], the 95% prediction intervals are calculated for each predicted value from the reduced models for the data points which are not used for fitting the model (Additional File 1, Section D).

#### Collinearity Analysis

For a least square regression approach, it is imperative for the residuals to be able to adhere to the assumption of constant variance. This property is also known as homoscedasticity. Two different measures are considered, namely, the Variance Inflation Factor and Breusch-Pagan test [40], for analyzing this property shared between different cofactors in the models (Additional File 1, Section E). This also ascertains that there is no significant evidence that the assumptions underlying the fitting and estimation methods are violated.

### Tools and software

Several tools and software are used for different statistical analysis and model fitting to the data. MS Excel was used for data handling. The model computation and verification of results was performed using MATLAB [45] and the R-package [46].

## Results

The RSM models and their modified forms are discussed below for two types of malaria incidence data - SPR and TMC values - in two cities of India - Chennai and Mangalore. The important independent variables (climate variables, population size, and previous incidence of the disease) are assessed using the step-wise model reduction process (described in Methods section), which offer better models as well as reliable predictions for these two cities.

### Models

#### SPR time series in Chennai

The SPR data for Chennai city covers the period, January 2002 - December 2004 (Figure 2(a)). Study of the autocorrelation of SPR values, at various lags ranging from 1 through 11, shows significant relationship for lag-one (Additional file 1, Section A, Figure S1(a) and Table S1). This observation is a reflection on the period of sporogyny (15 to 23 days at 20°C of Plasmodium development inside the mosquito) [47]. This justifies the consideration of 'SPR values at lag-one' as an independent variable in the model. The other independent variables, on which the response (SPR values) depends, are environmental factors, such as average temperature, average temperature-at-lag-one and rainfall. On Examination of the pair-wise linear correlations and corresponding *t*-statistics (Additional file 1, Section B, Tables S2 and S3) between these independent variables and SPR did not give any significant relationship, indicating the presence of non-linearity. Since rainfall is strongly related to humidity, the latter was not included in the model. Thus the independent variables selected for model formulation were: SPR-at-lag-one (SPR_-1_), average temperature (T), population (P), rainfall (R) and average temperature-at-lag-one (T_-1_). The non-linear relationship shared between the independent variables (except SPR-at-lag-one), and the SPR values, were confirmed using residual plots (Additional file 1, Section B, Figure S2). The correlation and residual plots of these indicate non-linear relationships, and the Second Order RSM model gave better initial fit than the First Order.

The model formulation using the Second Order model is then performed. The initial values of the parameters are optimized using response surface analysis. A salient feature of this system is the existence of a unique solution, which implies that each parameter can assume only one value, and hence, the initial solution space and optimized values of the estimated parameters overlap. The initial model with parameter estimates, based on the 80% of the SPR time series data of Chennai, is given by,

(4) $$\begin{array}{lll} \text{SPR} & =115.25-3.53\left(\text{SP}{\text{R}}_{-1}\right)-4.78\times 10^{-03}\text{T}\; & \text{(1)}\\ & -5.51\times 10^{-05}\text{P}+6.75\times 10^{-03}\text{R}+3.64\times 10^{-1}{\text{T}}_{-1}\; & \text{(2)}\\ & +10.07{\left(\text{SP}{\text{R}}_{-1}\right)}^{2}-3.61\times 10^{-04}{\text{T}}^{2}+6.52\times 10^{-12}{\text{P}}^{2}\; & \text{(3)}\\ & -3.48\times 10^{-07}{\text{R}}^{2}-1.63\times 10^{-03}{\left({\text{T}}_{-1}\right)}^{2}\; & \text{(4)}\\ & -8.35\times 10^{-02}\text{SP}{\text{R}}_{-1}\text{T}-3.48\times 10^{-07}\text{SP}{\text{R}}_{-1}\text{P}\; & \text{(5)}\\ & +1.09\times 10^{-03}\text{SP}{\text{R}}_{-1}\text{R}+8.83\times 10^{-02}\text{SP}{\text{R}}_{-1}{\text{T}}_{-1}\; & \text{(6)}\\ & +1.41\times 10^{-08}\text{T}\;\text{P}+2.34\times 10^{-05}\text{T}\;\text{R}\; & \text{(7)}\\ & +4.18\times 10^{-04}\text{T}\;\left({\text{T}}_{-1}\right)-1.57\times 10^{-09}\text{P}\;\text{R}\; & \text{(8)}\\ & -6.65\times 10^{-08}\text{P}\;\left({\text{T}}_{-1}\right)-2.02\times 10^{-05}\text{R}\;\left({\text{T}}_{-1}\right).\; & \text{(9)}\\ & \; & \text{(10)} \end{array}$$

**Figure 3:**
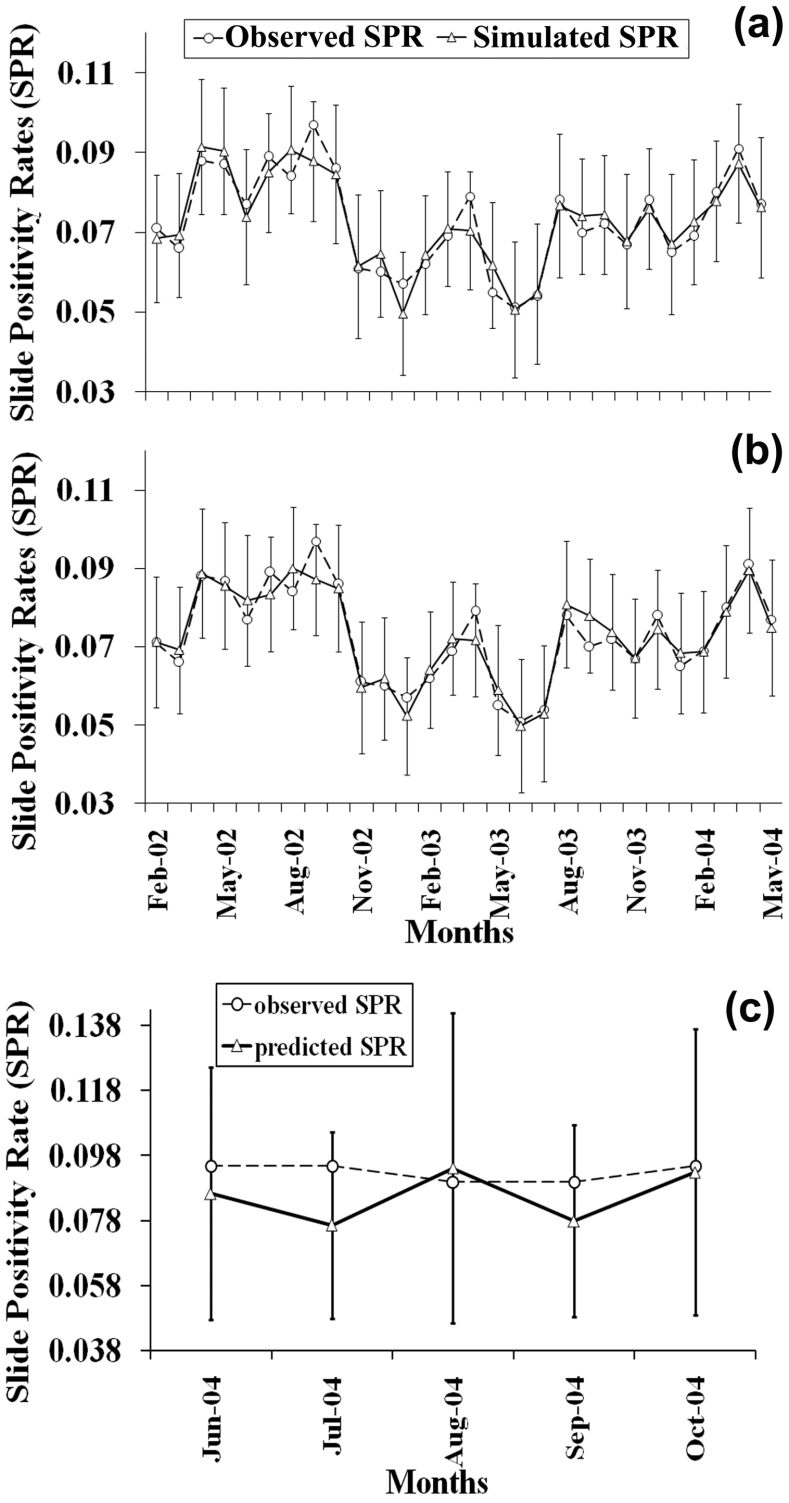
**The observed data and the model fit for SPR values in Chennai**. **(a) **Initial model using RSM; **(b) **Final model using model reduction techniques (Error bars show the 95% confidence intervals); and **(c) **Model validation using the final reduced model for SPR values in Chennai from June 2004 to October 2004 (Error bars show the 95% prediction intervals).

Figure 3(a) shows the actual time series data and the simulated SPR values from the initial model Eqn. (4). The lag variables ensure that every successive simulation (or estimation) provides an input to the model to obtain the next SPR value, capturing the autoregressive nature of the time series. The 95% confidence interval bars are shown in the plot of fit, which serve as error limits and validate the range of estimations. The estimations are considered reasonable when within the 95% confidence intervals. This model gives a coefficient of determination (R^2^) of 91.53%.

Next, the method of model reduction is followed, calculating the changed R^2 ^at each step and making the decision for removing or retaining a term accordingly (Additional file 1, Section C, Table S5). Being a manual and rigorous process, it is always possible to induct a term already removed if epidemiologically more important, though computationally it may have already been removed. The final model obtained using the above-mentioned process is given by,

(5) $$\begin{array}{ll} \text{SPR} & =75.96-20.40\left({\text{SPR}}_{-1}\right)-1.31\times 10^{-01}\text{T}\\ & -3.71\times 10^{-05}\text{P}+5.30\times 10^{-1}\left({\text{T}}_{-1}\right)\\ & +8.474{\left({\text{SPR}}_{-1}\right)}^{2}-1.07\times 10^{-04}{\text{T}}^{2}\\ & +4.46\times 10^{-12}{\text{P}}^{2}-1.21\times 10^{-03}{\left({\text{T}}_{-1}\right)}^{2}\\ & +4.4\times 10^{-06}{\text{SPR}}_{-1}\text{P}+3.28\times 10^{-08}\text{T}\;\text{P}\\ & +2.56\times 10^{-05}\text{T}\;\text{R}+2.402\times 10^{-04}\text{T}\;\left({\text{T}}_{-1}\right)\\ & -1.62\times 10^{-10}\text{P}\;\text{R}-1.04\times 10^{-07}\text{P}\;\left({\text{T}}_{-1}\right). \end{array}$$

The reduced model (Eqn.5) has R^2 ^= 89.75%. It is important to note that through this process it is possible to remove six terms from the initial model (about 30% reduction in coefficients) compromising only less than 2% decrease in the value of R^2^. Figure 3(b) shows the reduced model and fitted values along with the 95% confidence intervals computed for each time point. The estimated values are found to be consistently lying within the confidence intervals. This exercises a second level check on the efficiency of the model, in addition to the R^2^.

The AIC for the initial and final models for Chennai are - *3827.692 *and *3410.028*. It is clear that the reduced final model is indeed better owing to its lower AIC. For model validation the 20% of the SPR data (June to October 2004), which were not utilized for building the model, were estimated from the final model. Figure 3(c) shows the observed and predicted SPR values estimated from the final model of Chennai (Eqn. 5) along with the 95% prediction intervals.

#### TMC time series in Mangalore

The TMC data for Mangalore city covers 60 months, from January 2003 through December 2007 (Figure 2(c)). This dataset has an inherently different numerical nature from the SPR data. Based on the autocorrelation and correlation studies (Additional file 1, Section A, Table S1 and Figure S1(b)), the independent variables selected were: TMC-at-lag-one (TMC_-1_), average temperature (T), rainfall (R) and average temperature-at-lag-one (T_-1_). Also, even though the lag-two TMC variable (TMC_-2_) showed significant autocorrelation, it is not considered as an additional independent variable since the number of cases in the present month is considered to be directly influenced by the previous month only [47].

The linear correlation between TMC and the climate variables (Additional file 1, Section B, Table S4) were found to be significant. Here, 48 time points (80% of the data) were used to obtain the initial parameter estimates. The Second Order model gave better results, in contrast to the first order equation, owing to the non-linear relationships (Additional file 1, Section B, Figure S3). This system, too, has a unique solution which leads to an intersection between the initial estimates and the optimized estimates from response surface analysis. The initial model with parameter estimates is given by,

(6) $$\begin{array}{ll} \text{TMC} & =6995.79-1087.87\text{T}+4.03\text{R}-1.44{\text{TMC}}_{-1}\\ & +516.88\left({\text{T}}_{-1}\right)+40.03{\text{T}}^{2}-1.9\times 10^{-4}{\text{R}}^{2}\\ & -2.1\times 10^{-4}\;{\left({\text{TMC}}_{-1}\right)}^{2}+6.08{\left({\text{T}}_{-1}\right)}^{2}\\ & +5.68\times 10^{-2}\text{T}\;\text{R}-5.64\times 10^{-2}{\text{TMC}}_{-1}\text{T}\\ & -35.24\text{T}\;\left({\text{T}}_{-1}\right)-2.6\times 10^{-04}{\text{TMC}}_{-1}\text{R}\\ & -1.59\times 10^{-1}\text{R}\;\left({\text{T}}_{-1}\right)+1.67\times 10^{-1}{\text{TMC}}_{-1}{\text{T}}_{-1}. \end{array}$$

**Figure 4:**
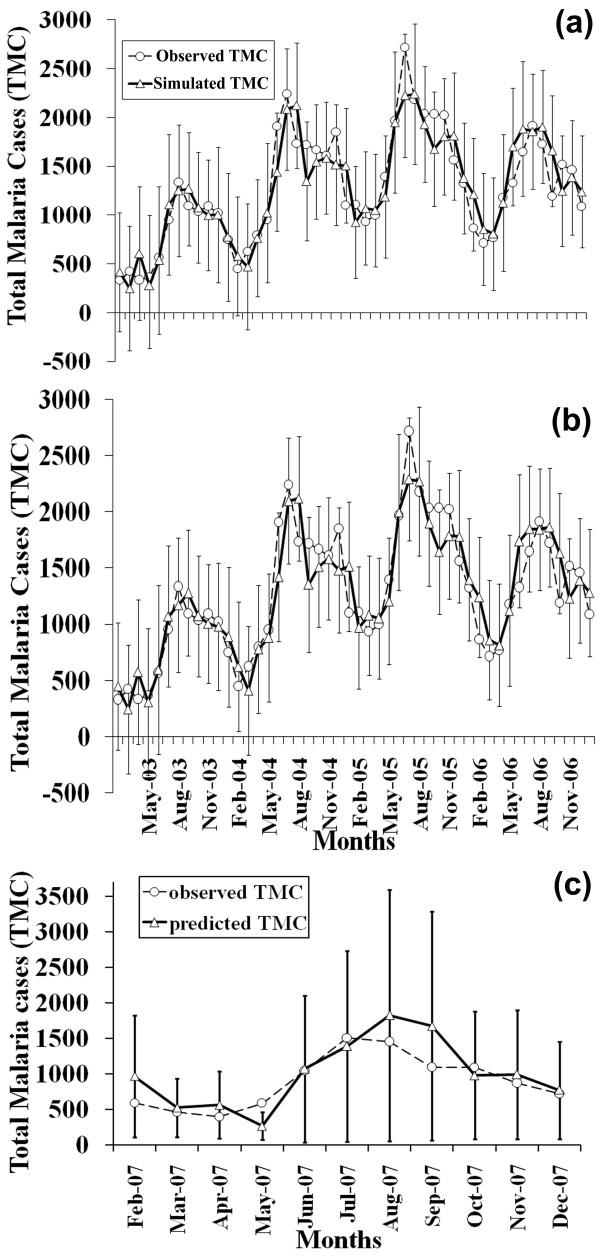
**The observed data and the model fit for TMC values in Mangalore**. **(a) **Initial model using RSM; **(b) **Final model using model reduction techniques (Error bars show the 95% confidence intervals); and **(c) **Model validation using the final reduced model for TMC values in Mangalore from February 2007 to December 2007 (Error bars show the 95% prediction intervals).

Figure 4(a) gives the initial model estimates of the response, TMC, and its observed values along with the 95% confidence limits. On applying the method of model reduction to equation (6), the final model with corresponding parameter estimates is given by,

(7) $$\begin{array}{ll} \text{TMC} & =9800.71-1077.33\text{T}+3.43\text{R}-2.30{\text{TMC}}_{-1}\\ & +348.61\left({\text{T}}_{-1}\right)+18.64{\text{T}}^{2}-2.2\times 10^{-4}{\left({\text{TMC}}_{-1}\right)}^{2}\\ & +9.958{\left({\text{T}}_{-1}\right)}^{2}-4.378T\;\left({\text{T}}_{-1}\right)-1.1004\text{R}\;\left({\text{T}}_{-1}\right)\\ & +1.40\times 10^{-1}{\text{TMC}}_{-1}{\text{T}}_{-1}. \end{array}$$

This process reduces the number of terms from 15 to 11 (~27% reduction), and hardly reduces the R^2 ^from 84.61% to 84.28% (only by 0.4%). Further attempts to remove terms (Additional file 1, Section C, Table S6) resulted in drastic reductions in the value of R^2^. Figure 4(b) shows the observed and estimated values for the final reduced model. In this case also, the AIC can be computed for the initial as well as the final model, which are - *3812.048 *and *3359.722*. The lower AIC value for the final model proves that this parsimonious representation of the same information with lower number of terms is a useful approach to model epidemiological data. For model validation TMC for Mangalore from February to December 2007 were considered. Figure 4(c) shows the observed and predicted TMC values estimated from the final model of Mangalore (Eqn. 7) along with the 95% prediction intervals.

Moreover, to assess the linear accuracy, homoscedasticity and to observe the differences after removing lower order variables, residual plots for the initial and reduced models have been plotted for both SPR and TMC values (Additional file 1, Section C, Figure S5), which show uniform spread of the residual cloud indicating homoscedasticity.

## Discussion

In this study, a combination of statistical modelling approach (the RSM) and a simple model reduction method is applied to describe the incidence of malaria in two geographically, ecologically and demographically different cities in India (Chennai, Tamil Nadu and Mangalore, Karnataka) for two types of epidemiological data - Slide Positivity Rates (SPR) values of malaria for Chennai and Total Malaria cases (TMC) for Mangalore. This approach shows the applicability of the algorithm to different measures of incidence of malaria. The analyses of the parasitological data for the two Indian cities using the RSM approach not only capture the essential dynamics of the disease incidence, but also show the influence of different climatic and non-climatic factors. For both Chennai and Mangalore city, it is clear from the final model equations that previous incidence of the disease, Temperature as well as Temperature-lag-one and Rainfall have strong influence on the disease incidence and play important roles to shape the disease curve. The predictions for Chennai city do not show any clear trend for the disease to decrease but show more monthly periodic variations. But for Mangalore city, the prediction show initial decay in the total malaria cases from February to May, then show an upward trend till August, and finally decreasing again till December following the pattern of rainfall distribution of that region.

RSM is a global approximation method which is ideally suited for solving problems with a relatively noisy response, where a gradient based method would lead to a local optimum instead of a global one [32]. The Second Order model given by the RSM approach, in spite of not having terms higher than degree 2, still represents the temporal variations of both datasets very well. Further, there is no linearity assumption in the analysis. The previous incidence of the disease and the influence of previous temperature as independent variables are also introduced. This is an 'auto correlative concept' that ensures training of the model on past values and leads to autoregressive forecasts by the model, prediction of future incidence and, thus, establishing the applicability of RSM to statistical modeling of epidemiological data.

A model reduction method is introduced, which ensures filtering of terms that do not make valuable contribution to the fit of the model. It is a simple but useful approach, and has no underlying assumption except that the contribution of a term in the model is directly proportional to its order of magnitude. Based on this, a criterion to remove terms from the model without a significant change in the coefficient of determination is successfully outlined. At each step of the model reduction, it is ensured that the predictions remain within the confidence limits that minimize error variance. It is worth noting here that the proposed model reduction technique simplifies the model structure by reducing number of coefficients (terms) by almost 30% compared to the initial model by RSM without reducing the coefficient of determinant values significantly (around 0.4-2% decrease in R^2^).

The results based on the initial model by RSM, and final model through the proposed model reduction technique, show good fits for both the data types in two different cities (Figures 3 and 4). It may be worth mentioning that the R^2 ^in this study for the SPR values in Chennai city is much higher (89.75%) compared to previously reported values (63.85%) observed on the same data using a different regression method [28]. Using Akaike Information criterion (AIC), it is successfully established that the reduced models for both cities are the best fit models. The observed and estimated values lie within the 95% confidence limits. Also, the forecasts lie within the 95% prediction intervals (Figures 3(c) and 4(c)). Thus, the reduced models perform well and are useful for forecasting purposes, provided reliable climate variable estimates and population projections are available. The chi-square goodness of fit is also found to be highly statistically significant (p-value < < 0.001), which gives another affirmative test, in addition to the confidence limits, AIC and prediction limits, thereby rendering significantly good comparability between the observed and predicted values. From the residual plots for both SPR as well as TMC values (Additional File 1, Figure S5), it is observed that there is slight compression of the residual cloud from the initial to the reduced model for SPR as well as TMC, but the spread in general is uniform on either side of the x-axis confirming homoscedasticity. To explore the nature of the residuals and further to measure the collinearity imposed by different cofactors in the models, criteria such as, Variance Inflation Factor and Breusch-Pagan test have been used. It is observed that the chosen independent variables in both data, do not lead to violation of the assumption of constant variance of the residuals (Additional File 1, Table S7 (a) and (b)) and the homoscedastic null hypothesis is not rejected at 99% level of significance (Additional File 1, Table S8). This reinstates that the residuals show no erratic behavior as a function of the value of the responses, and hence it can be assumed that there is no significant level of heteroscedasticity. This provides a comprehensive reason for the use of our model fitting approach for the available data.

The comparison of initial and final model formulations (Eqns. 4 and 5) for the SPR values in Chennai city reveals some interesting features. The final model not only contains less number of dependent terms but also shows reduction of direct dependence of both linear and higher order terms for rainfall. The dependence of SPR on rainfall in this final model (Eqn. 5) is only depicted by the interaction terms associated with other factors, such as temperature and population size. This feature is also observed in the original data (Figure 2(a) and 2(b)) as the variation in SPR values do not follow exactly the rainfall distribution in this region, which clearly has two peaks every year due to North-East and South-West monsoons. Moreover, the ups and downs of the SPR values, whatever was observed in the real data, follow the temperature distribution of this city broadly. This pattern is also captured by the RSM model, which shows more dependence of SPR values on temperature, either through direct presence of linear and higher order terms, or through interactions with other associated factors including rainfall. The presence of interaction terms and non-linear terms enables proper representation of the nature of SPR values of Chennai. On the other hand, the Total Malaria cases for Mangalore follows a cyclic pattern each year with gradual increase leading to the peak during June - August, followed by a gradual decrease, quite similar to the rainfall distribution in this region due to the direct influence of the Arabian Sea branch of the South-West monsoon (Figures 2(c) and 2(d)). This specific trend is also mimicked by the model proposed here using the combined RSM and model reduction technique. The model equation (Eqn. 7) shows direct dependence on rainfall term and captures the essential dynamics of the disease along with other factors. Another interesting observation in this study is the presence of less direct terms (linear or higher-order) and more cross interaction terms in Chennai model compared to Mangalore model. This also highlights the complex influence of environmental variables on the epidemiological status of these regions. Chennai being in the tropical wet-dry climatic region shows sustained prevalence of malaria through-out the year with less periodic variations, and hence the variations in the SPR values are associated with more cross interaction terms. Whereas, Mangalore being in tropical dry climatic region shows more periodic variations in TMC values following the climatic changes in the region, which are observed to be directly influencing the model formulation.

Since the observed and estimated values do compare well, we would like to infer that these techniques of modelling (the RSM and the model refinement/reduction method) perform well for analyzing epidemiological data and may be useful for forecasting purpose, provided reliable climate variable estimates and population projections are available.

## Conclusions

Mathematical analysis of the models may not always capture the essential features of the disease transmission process, as it is sometimes difficult to understand the direct and interacting effects of temperature, seasonal forcing, excessive rainfall, correlation between different variables and other model parameters. Also, among the innumerable statistical models based on malaria incidence data, only a few approaches have shown promising results. The results of these models are mostly data specific, and applicable primarily to the particular data set studied. Therefore, there is always a need for suitable models and methods to understand the important features of the epidemiological data for providing better predictions.

In this paper the Response Surface Method (RSM) is applied for the first time in analyzing epidemiological data, specifically for malaria. It has been shown here that this method models the existing time series data well. The model is subjected to further analysis and development of a simpler, but statistically good, reduced model, which can offer better forecast of the disease. Since the major challenge is to ascertain the detailed understanding of the different environmental factors, previous incidence of the disease and their influence on the prevalence pattern, a reduced model with fewer terms is helpful for clearer understanding and reliable predictions.

The interesting effects of climatic and non-climatic factors, such as previous disease incidence, population, are clearly visible from the analysis. The model predictions capture the climatic variations, mainly rainfall and temperature, for both the regions under study and resembles well with the observed disease incidence. This approach leads to detection of the most crucial environmental factors influencing the transmission of the disease while offering a coherent and integrated understanding of the disease process in any area. Thus, the proposed combined method gives a simple, but highly predictive model for malaria incidence without compromising on the proportion of variation represented and may be useful for public health professionals for adopting better strategy not only to control malaria but for other infectious diseases, if suitable climatic information and disease prevalence data are available.

## Competing interests

The authors declare that they have no competing interests.

## Authors' contributions

SBR carried out the theoretical analysis, and contributed in writing the manuscript. RRS contributed in theoretical analysis and formulating the approach, and writing of the manuscript. SS conceived the idea for the article, and contributed to designing the study and writing of the manuscript. All authors read and approved the final manuscript.

## Supplementary Material

Additional file 1 **Statistical techniques and Model Reduction Process**. The additional information provided describe the statistical techniques used for exploring the relationships between dependent variables (SPR and TMC values) and independent variables (environmental factors, previous incidence of the disease etc.) and the details of the model reduction process (also includes Tables S1-S8 and Figures S1-S5). [40-42]. Click here for file
