# Supplementary material for: Theoretical investigation of malaria prevalence in two Indian cities using the response surface method
Source: Malar J. 2011 Oct 14;10:301. doi: 10.1186/1475-2875-10-301 (PMC3224354; doi:10.1186/1475-2875-10-301)
Supplement: Additional file 1 — Statistical techniques and Model Reduction Process. The additional information provided describe the statistical techniques used for exploring the relationships between dependent variables (SPR and TMC values) and independent variables (environmental factors, previous incidence of the disease etc.) and the details of the model reduction process (also includes Tables S1-S8 and Figures S1-S5). [40-42]. [file 1475-2875-10-301-S1.PDF]

Additional File: Statistical techniques and Model Reduction Process

Section A

Autocorrelation: The autocorrelation is studied at different lags for SPR and TMC values for Chennai and Mangalore, respectively (Figures S1 a, b). This provides us the relation between the present values of variables with their values at a previous time point [40]. The figures show the 95% confidence limits for the autocorrelation values at lags from 1 through 11. Due to the periodic pattern of the data, it is expected that for larger lag the pattern would repeat itself. It is possible to observe, in general, that the autocorrelation at lag-one is significant as it is far beyond the error limits. Hence, SPR-at-lag-one (SPR_{-1}) is considered as an additional independent variable for Chennai, while TMC-at-lag-one (TMC_{-1}) is included among the independent variables for Mangalore. But, for TMC, in addition to the lag one value, the value at lag two, also, is greater than the 95% limits permit. However, this behaviour is neglected, since it is expected that the number of cases in the previous month would affect the current month's number of cases more than those in two months before. The same pattern is repeated for larger lags.

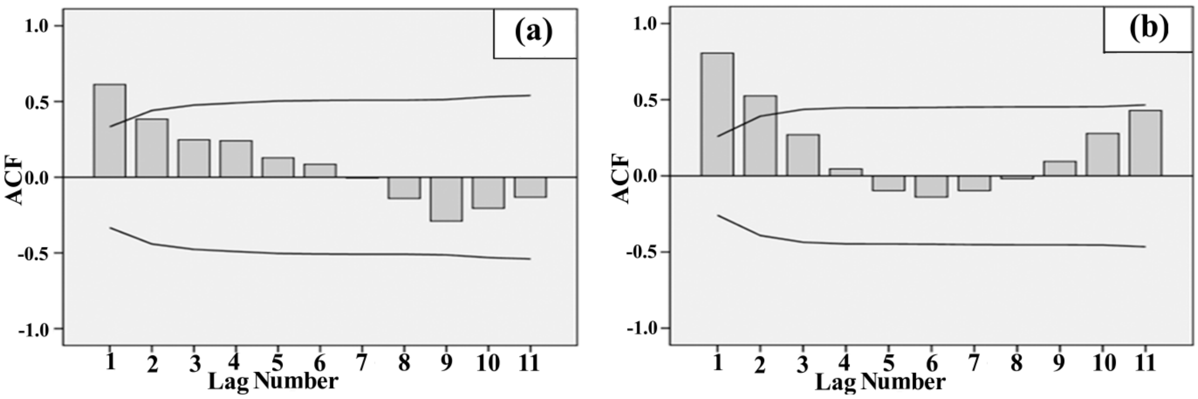

***Figure S1: Autocorrelation plots**. **a)** SPR values of Chennai from January 2002 to December 2004; and **b)** TMC of Mangalore from January 2003 to December 2007, all at Lags 1 through 11. The lines are for 95% confidence limits.*

Ljung-Box Statistic: This statistic gives the significance of autocorrelation at different lags, cumulatively [40]. It shows highly significant *p*-values at lag one (Table S1). Hence, the observations from autocorrelation plots can be verified through this statistic. The significance of the statistic at lag one is carried over to some of the *p*-values for the following few lags since it is a cumulative measure.

Table S1 *Ljung-Box statistic for SPR values of Chennai and TMC values of Mangalore with p-values at 95% level of significance*

**Lag↓**	**ACF_1**	**LB_1**	**p_1**	**ACF_2**	**LB_2**	**p_2**
-1	0.613681	14.34421	0.000152	0.819723	38.96123	0.0001
-2	0.387528	20.23755	0.00004	0.535942	55.90804	0.0001
-3	0.285586	23.53816	0.000031	0.260731	59.99051	0.0266
-4	0.284155	26.91116	0.0205	0.013578	60.00178	0.0052
-5	0.147157	27.84595	**0.1671**	-0.14507	61.31252	**0.2084**
-6	0.115245	28.43903	**0.3014**	-0.18232	63.42165	**0.8515**
-7	-0.01087	28.44449	**0.9142**	-0.12218	64.38713	**0.5598**
-8	-0.19157	30.20476	**0.1287**	-0.0451	64.52124	0.0231
-9	-0.45511	40.52099	0.0003	0.083952	64.99529	0.0142
-10	-0.37242	47.70555	0.0043	0.31126	71.6446	**0.9259**
-11	-0.25344	51.17141	**0.0516**	0.503997	89.44137	0.0002

Suffix 1 indicates the value corresponds to SPR values; Suffix 2 indicates the value corresponds to TMC values; ACF: autocorrelation function; LB: Ljung-Box statistic; *p*: *p*-value.

Note: p-values in bold face are not significant

Section B

Correlation: The correlations are studied by turns between the response variable (SPR or TMC as the case may be) and each of the corresponding independent variables (Tables S2 - S4). For each pair of response variable and independent variable (say, SPR and SPR_{-1}), values from the beginning (denoted by the '+' sign) or from the end (denoted by the '-' sign) of the series are systematically deleted and the pair-wise correlations (given in the column labeled with the corresponding independent variable) with the accompanying *p*-value (given in the columns labeled '*p*') are calculated. This helps in exploring whether the correlation shown is consistent and real when all the observations are considered, lending informed insight into the linear relationships between the variables in the model [41]. Apart from SPR and SPR_{-1}, and also, TMC and TMC_{-1}, the correlation values of the other variables are not very significant, though they are known to be influential factors. One possible reason for this is due to use of linear correlation measure to capture the relationship between dependent and independent variables. This further indicates that the non-linearity of the relations needs to be studied to explore the real relationship among the variables.

Table S2 *Correlation between SPR values (for Chennai) and SPR-at-lag-one, Population, Average Temperature with p-values at 95% level of significance*

**Number of values deleted from the beginning (+) or end (-) of the data series ↓**	**SPR_{-1}**	***p***	**T**	***p***	**P**	***p***
**-11**	0.526	0.004	0.194	**0.181**	-0.398	0.027
**-10**	0.527	0.003	0.199	**0.17**	-0.389	0.027
**-9**	0.516	0.003	0.198	**0.166**	-0.319	**0.056**
**-8**	0.528	0.002	0.241	**0.113**	-0.194	**0.167**
**-7**	0.523	0.002	0.245	**0.105**	-0.162	**0.205**
**-6**	0.516	0.002	0.285	**0.067**	-0.044	**0.41**
**-5**	0.563	6×10^{-04}	0.309	0.048	0.0519	**0.393**
**-4**	0.588	3×10^{-04}	0.332	0.034	0.1132	**0.272**
**-3**	0.606	1×10^{-04}	0.328	0.033	0.1663	**0.182**
**-2**	0.626	5×10^{-05}	0.294	0.049	0.2286	**0.1**
**-1**	0.642	2×10^{-05}	0.255	**0.073**	0.2703	**0.061**
**0**	0.616	4×10^{-05}	0.263	**0.063**	0.2389	**0.083**
**1**	0.614	6×10^{-05}	0.256	**0.072**	0.2297	**0.096**
**2**	0.611	8×10^{-05}	0.256	**0.075**	0.2006	**0.132**
**3**	0.645	3×10^{-05}	0.246	**0.087**	0.2598	**0.076**
**4**	0.638	6×10^{-05}	0.221	**0.116**	0.3212	**0.039**
**5**	0.642	7×10^{-05}	0.222	**0.119**	0.3421	**0.032**
**6**	0.65	7×10^{-05}	0.19	**0.162**	0.4247	0.011
**7**	0.644	1×10^{-04}	0.177	**0.183**	0.4904	0.004
**8**	0.643	1×10^{-04}	0.156	**0.218**	0.6513	1×10^{-04}
**9**	0.63	3×10^{-04}	0.169	**0.205**	0.7591	3×10^{-06}
**10**	0.684	8×10^{-05}	0.133	**0.263**	0.7518	7×10^{-06}
**11**	0.671	2×10^{-04}	0.072	**0.37**	0.7387	2×10^{-05}

SPR_{-1}: S.P.R.-at-lag-one; T: Average Temperature; P: Population; *p*: *p*-value;

Note: p-values in bold face are not significant

Table S3 *Correlation between SPR values (for Chennai) and Rainfall, Average Temperature-at-lag-one with p-values at 95% level of significance*

**Number of values deleted from the beginning (+) or end (-) of the data series ↓**	**R**	***p***	**T_{-1}**	***p***
**-11**	0.13	0.273	0.1687	0.215
**-10**	0.135	0.26	0.1742	0.202
**-9**	0.124	0.273	0.1339	0.257
**-8**	0.124	0.268	0.1321	0.256
**-7**	0.129	0.257	0.142	0.236
**-6**	0.128	0.255	0.159	0.205
**-5**	0.087	0.324	0.2024	0.142
**-4**	0.122	0.256	0.2249	0.112
**-3**	0.122	0.254	0.2505	0.083
**-2**	0.187	0.148	0.248	0.082
**-1**	0.251	0.076	0.2272	0.098
**0**	0.258	0.067	0.2341	0.088
**1**	0.252	0.076	0.2253	0.1
**2**	0.235	0.094	0.2052	0.126
**3**	0.267	0.07	0.2104	0.124
**4**	0.282	0.062	0.2005	0.14
**5**	0.287	0.062	0.203	0.141
**6**	0.31	0.051	0.1771	0.051
**7**	0.302	0.059	0.1557	0.214
**8**	0.304	0.061	0.1252	0.267
**9**	0.267	0.094	0.1122	0.293
**10**	0.417	**0.019**	0.1017	0.314
**11**	0.408	**0.024**	0.055	0.399

R: Rainfall; T_{-1}: Average Temperature-at-lag-one; *p*: *p*-value;

Note: p-values in bold face are significant here.

Table S4 *Correlation between different independent variables and TMC (for Mangalore) with p-values at 95% level of significance*

**Number of values deleted from the beginning (+) or end (-) of the data series ↓**	**T**	***p***	**R**	***p***	**TMC_{-1}**	***p***	**T_{-1}**	***p***
**-11**	-0.621	1×10^{-06}	0.402	0.002	0.8244	1×10^{-07}	-0.415	0.002
**-10**	-0.606	2×10^{-06}	0.413	0.002	0.819	0.001	-0.393	0.003
**-9**	-0.618	1×10^{-06}	0.423	0.001	0.8246	1×10^{-07}	-0.38	0.003
**-8**	-0.638	1×10^{-07}	0.431	8×10^{-04}	0.8314	$1v10^{-07}$	-0.399	0.002
**-7**	-0.648	1×10^{-07}	0.434	7×10^{-04}	0.8357	0.001	-0.422	9×10^{-04}
**-6**	-0.642	1×10^{-07}	0.394	0.002	0.832	1×10^{-07}	-0.422	8×10^{-04}
**-5**	-0.644	1×10^{-07}	0.398	0.001	0.8268	1×10^{-06}	-0.425	7×10^{-04}
**-4**	-0.64	1×10^{-07}	0.401	0.001	0.8273	1×10^{-07}	-0.428	6×10^{-04}
**-3**	-0.628	1×10^{-07}	0.391	0.001	0.8238	1×10^{-07}	-0.413	8×10^{-04}
**-2**	-0.624	1×10^{-07}	0.392	0.001	0.8239	1×10^{-07}	-0.403	9×10^{-04}
**-1**	-0.618	1×10^{-07}	0.397	0.001	0.8232	2×10^{-05}	-0.395	0.001
**0**	-0.613	1×10^{-07}	0.404	8×10^{-04}	0.8242	1×10^{-07}	-0.387	0.001
**1**	-0.613	1×10^{-07}	0.393	0.001	0.8197	1×10^{-07}	-0.407	8×10^{-04}
**2**	-0.598	1×10^{-06}	0.382	0.002	0.8121	1×10^{-07}	-0.401	1×10^{-03}
**3**	-0.569	2×10^{-06}	0.373	0.002	0.8036	3×10^{-07}	-0.374	0.002
**4**	-0.537	1×10^{-05}	0.36	0.003	0.7929	1×10^{-07}	-0.325	0.008
**5**	-0.553	7×10^{-06}	0.475	1×10^{-04}	0.7847	5×10^{-07}	-0.281	0.02
**6**	-0.581	3×10^{-06}	0.512	4×10^{-05}	0.7851	1×10^{-06}	-0.286	0.019
**7**	-0.584	3×10^{-06}	0.514	5×10^{-05}	0.7896	1×10^{-07}	-0.289	0.019
**8**	-0.592	2×10^{-06}	0.513	6×10^{-05}	0.7912	1×10^{-07}	-0.297	0.017
**9**	-0.595	3×10^{-06}	0.512	7×10^{-05}	0.7904	1×10^{-07}	-0.306	0.015
**10**	-0.594	3×10^{-06}	0.509	9×10^{-05}	0.7897	1×10^{-06}	-0.308	0.016
**11**	-0.608	2×10^{-06}	0.506	1×10^{-04}	0.7888	1×10^{-07}	-0.303	0.018

T: Average Temperature; R: Rainfall; TMC_{-1}: TMC-at-lag-one; T_{-1}: Average Temperature-at-lag-one; *p*: *p*-value;

Note: All p-values are significant here.

Residual Plots (Preliminary Data): The residual plots are graphical representations of the differences between the fitted and the observed values of responses [41]. Here, residuals are plotted for linear models for each independent variable with the appropriate response, SPR or TMC values (Figures S2, S3). If the independent variable is indeed related to the response linearly, then the spread of residuals must be uniform or random in the x-y plane. This should be the case when the corresponding correlation was found significant. If not, that is, if the relationship is non-linear (linear correlation is not significant), the residuals must show a non-random pattern. For instance, in Figure S2(a) for Chennai, the plot for SPR_{-1} is well-distributed with more or less equal number of points on either side of the x-axis, whereas, for T, the same plot becomes skewed with a visible funnel formation which indicates the non-linearity in the relation it shares with SPR (Figure S2(b)). Similar observations are made for the other independent variables, Population (P), Rainfall (R) and Average Temperature at lag one (T_{-1}) (Figure S2(c-e)). This indicates the non-linear relationship among these variables with the response variable, which was not observed in the linear correlation measure performed earlier. For Mangalore also it is observed that non-linearity prevails for the independent variables, Average Temperature (T), Rainfall (R), and Average Temperature-at-lag-one (T_{-1}), with the response variable TMC values (Figure S3 (a, b and d)), whereas TMC-at-lag-one (TMC_{-1}) is well-distributed with more or less equal number of points on either side of the x-axis depicting linear relationship with TMC (Figure S3(c)).

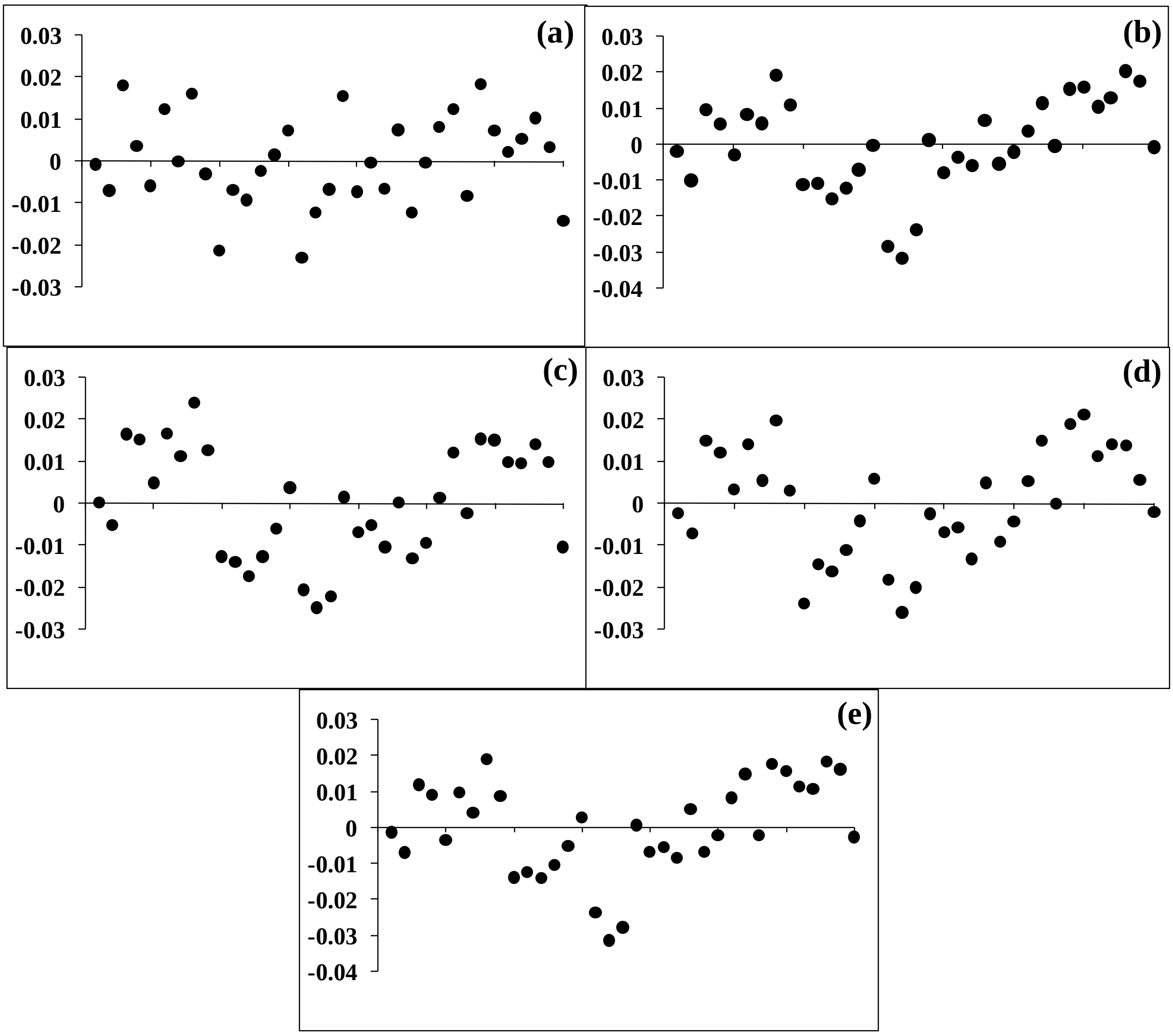

Figure S2: Residual plots for the dependent variable, SPR values of Chennai from January 2002 to December 2004, with the independent variables. *(a) SPR-at-lag-one:SPR$_{-1}$; (b) Average Temperature:T; (c) Population:P; (d) Rainfall:R; and (e) Average Temperature-at-lag-one:T$_{-1}$*

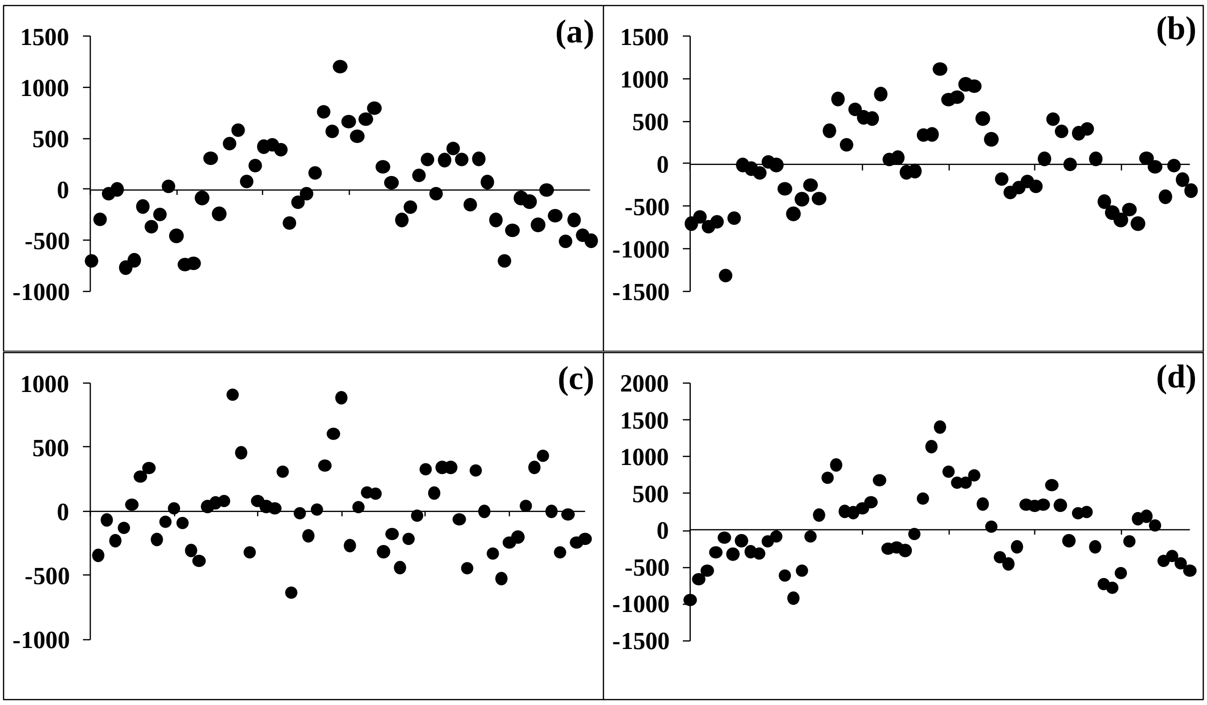

Figure S3: Residual plots for the dependent variable, TMC values of Mangalore from January 2003 to December 2007, with the independent variables*. (a) Average Temperature:T; (b) Rainfall:R; (c) TMC-at-lag-one:TMC*$_{-1}$*; and (d) Average Temperature-at-lag-one:T*$_{-1}$

Section C

Model development using Response Surface Method

Response Surface Method is mostly been employed when experiments are conducted by varying the levels of the different factors and observing the effect on the response, training the values in a manner so as to reach the optimum point on the response surface [42]. The first step in RSM is to find the First Order model with parameters $\beta_1, \beta_2, \dots, \beta_k$, which is given by

$$Y = \beta_o + \sum_{i=1}^{k} \beta_i X_i + \epsilon., \quad \text{(A1)}$$

where Y is the response variable and X_1, X_2, X_3, …, X_k is the set of k independent variables. If the preliminary analysis indicates non-linearity in the relation between independent variables and the response, along with the linear terms, a quadratic polynomial is used to include, the second order terms of the independent variables individually and all $\binom{k}{2}$ possible interaction terms of order 2. Hence, the Second Order model is given by

$$Y = \beta_o + \sum_{i=1}^{k} \beta_i X_i + \sum_{i=1}^{k} \beta_{ii} X_i^2 + \sum_{i<j} \beta_{ij} X_i X_j + \epsilon. \quad \text{(A2)}$$

The method of least squares is then applied to obtain an initial estimate of the parameters for the First or Second Order model depending on the cases. The response surface analysis is then performed, using the fitted surface to obtain the values of the parameters that optimize the response value. Once the independent variables are chosen using the methods mentioned in the above two sections, the required model is fitted using this RSM method. Due to non-linear relations, the second order model is used, which includes the linear terms, individual quadratic terms and all possible second degree interaction terms for the independent variables.

Model Reduction Process: Further, a systematic model reduction process is implemented to derive a simpler model with fewer but important terms, while ensuring efficiency (coefficient of determination) similar to that obtained in the initial model fitting through RSM. Fig. S4 shows the flow chart of the systematic process followed for model development using RSM and the model reduction technique.

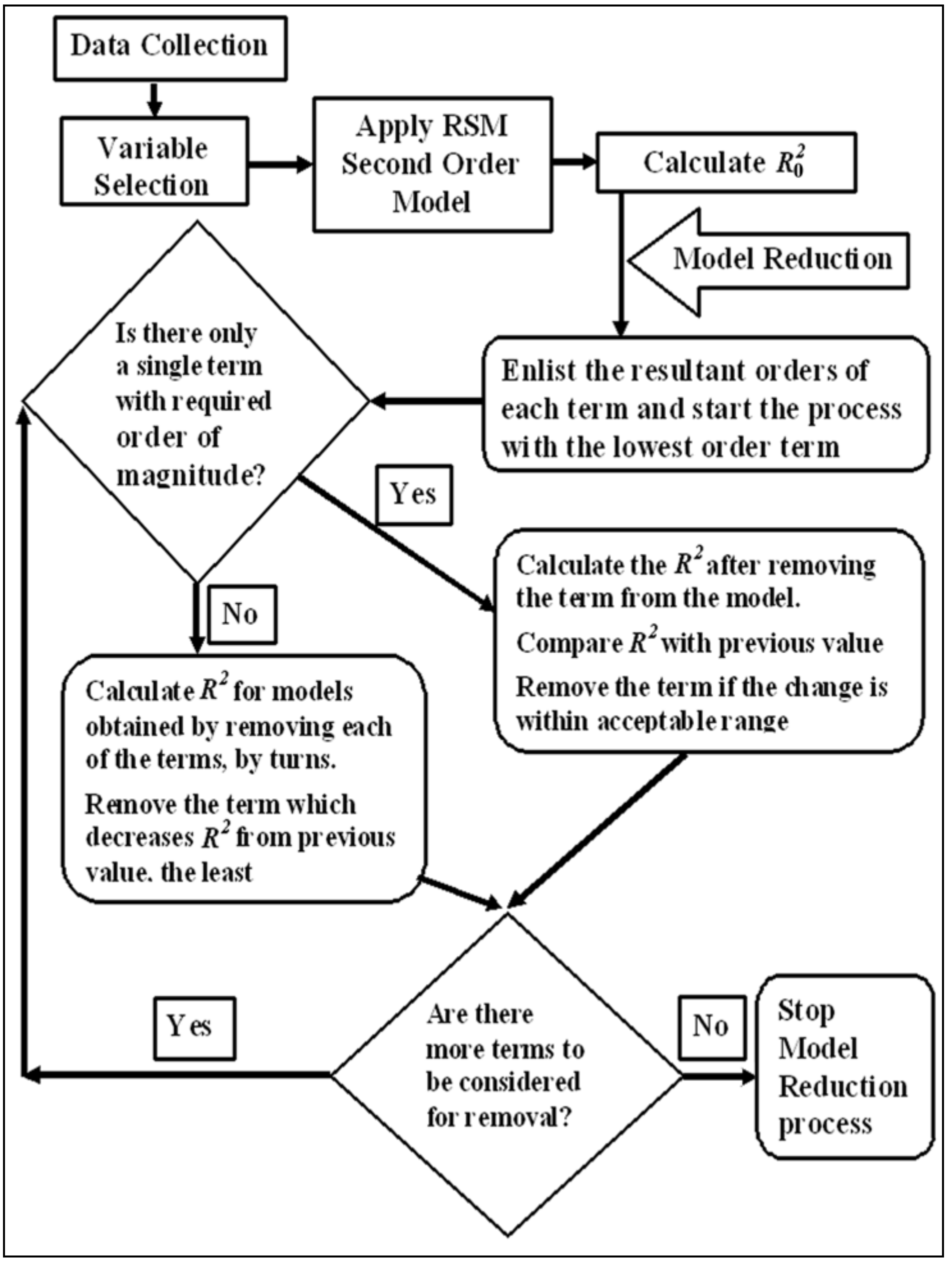

Figure S4: Flowchart for model development using Response Surface Method and Model Reduction process. *R_0^2 denotes the coefficient of determination for the initial model obtained using only RSM.*

In this process, first the resultant orders of magnitude of each of the terms (for instance, 56.789 = 5.6789×10^1 has order 1 while 0.56789=5.6789×10^{-1} has order -1) in the initial model are enlisted. Each term consists of two components: the variable – linear or quadratic (for instance, SPR values are of the order of -2 while its quadratic term will have an order of -

4) and the corresponding estimated coefficient or parameter value. Once the order of the variable and the associated parameter or coefficient in the second order fitted model equation is known, the resultant order of each member can be obtained by simply adding the corresponding contributing orders (for instance, SPR values have order -2 and if its coefficient has an order of 1, then the resultant order of this member will be -2+1 = -1). The constant in the equation has not been considered in this analysis.

Following the above process, the coefficient of determination of the model is examined by removing each term successively, starting with the term with the smallest resultant order. Next, the value as well as the change in the coefficient of determination or R^2 of the model are examined when a term is removed from the model (Tables S5 -S6) at each step. The change will always be negative under realistic conditions. The decision for retaining or removing a variable from the model varies from one case to another. This process is followed stepwise, considering removal of terms with successively higher resultant orders. At each step, the relative change in the coefficient of determination is considered from the previous step. If there is no change, this is continued without retaining the removed term in the model. If the change is negative, then, the criterion used is considering the scale of change. In case the change is in the second, third or fourth decimal, it is allowed and the subsequent step is followed. If not, then it is implied that the removed term makes important contribution to the value of response. Hence, that term will continue to remain in the model. Also, positive change is not mentioned, since it is highly improbable that removal of a term from a model would increase its coefficient of determination. Further, in case of terms with tied resultant orders, those with the same order have been considered for removal, in turns, in the same step, and the removal of the term that leads to the least negative change in the coefficient of determination is retained. In this work, a term is retained if the change in R^2 is in the first decimal place, else, it is removed.

Table S5 *Model Reduction Steps for the Second Order Response Surface model for SPR values of Chennai*

	Terms Removed	R-square	Decision
**Step 1**	X4^2	0.9118	Remove
**Step 2**	X1*X4	0.909	Remove
**Step 3**			
3a	X2*X4	0.9054	Retain
3b	X4*X5	0.9089	Remove
**Step 4**			
4a	X4	0.8989	Remove
4b	X3*X4	0.8956	Retain
**Step 5**			
5a	X1^2	0.8937	Retain
5b	X1*X2	0.8989	Remove
5c	X1*X3	0.8954	Retain
5d	X1*X5	0.8984	Retain
**Step 6**			
6a	X1*X3	0.895	Retain
6b	X1*X5	0.8975	Remove

X1: SPR-at-lag-one or SPR_{-1}; X2: Average temperature or T;

X3: Population or P; X4: Rainfall or R; X5: Average Temperature-at-lag-one or T_{-1}

Table S6 Model Reduction Steps for the Second Order Response Surface model for TMC values of Mangalore*

	**Terms Removed/Added**	**R-square**	**Decision**
**Step 1**	X2^2	0.8456	Remove
**Step 2**	X2*X3	0.8439	Remove
**Step 3**	X1*X2	0.8429	Remove
**Step 4**			
4a	X2*X4	0.8411	Remove
4b	X2	0.8401	Retain
**Step 5**	X1*X3	0.8411	Remove
**Step 6**	Add X1*X2	0.8428	Add (epidemiologically important, better fit)

X1: Average Temperature or T; X2: Rainfall or R; X3: TMC-at-lag-one or TMC_{-1}; X4: Average Temperature-at-lag-one or T_{-1};

In all the above cases the estimation of coefficients (parameters) for each term changes accordingly at each step. This process is continued till terms with resultant orders equal to that of the response or higher are reached. Here, the underlying assumption is the contribution of a term to a model is directly proportional to its order of magnitude. But, only those members are considered, which have resultant orders less than that of the response variable since the terms with orders equal to or higher than that of the response are expected to make very important contribution to the quality of model fit and prediction. Also, being a manual procedure, it is possible to re-induct terms that have been removed due to its cited

biological importance. Hence, this procedure finally leads us to a relatively parsimonious model that still explains a sizeable proportion of variation in the model.

In order to gauge the differences in fits of the initial models and reduced models, their respective residual plots for both SPR as well as TMC values (Figure S5) are considered. There is slight compression of the residual cloud from the initial to the reduced model for SPR (Figure S5 (a), (b)) as well as TMC (Figure S5 (c), (d)). The spread in general is uniform on either side of the x-axis which indicate homoscedasticity.

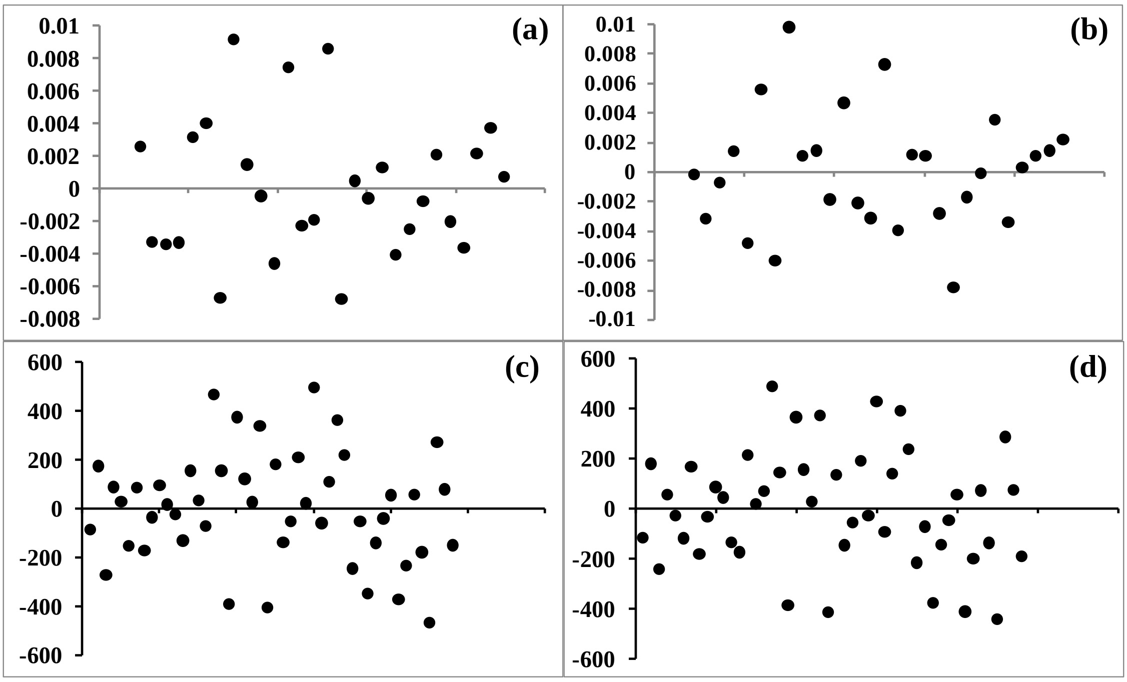

Figure S5: Residual plots. *For the dependent variable, SPR values of Chennai from January 2002 to December 2004: (a) Initial model; (b) Model after Reduction; For response variable, TMC values of Mangalore from January 2003 to December 2007: (c) Initial model; and (d) Model after Reduction*

Section D

Confidence intervals for the fitted response obtained from the initial models and *prediction intervals* for the estimated future values from the reduced models are constructed.

Construction of Confidence Intervals: Let the response vector *Y* consist of *n* values, $y_1, y_2, \dots, y_n$ and it has an error related to it which is assumed to be a white noise with zero mean and constant variance. Let there be independent variable matrix $X_1, X_2, \dots, X_k$, which is considered to be error-free. Then there will be parameters, $\beta_1, \beta_2, \dots, \beta_k$, such that the model is given by formula (A3).

$$y_i = \sum_{j=1}^{k} X_{ij}\beta_j + \epsilon_i, \tag{A3}$$

where, ϵ_i 's are $N(0,\sigma^2)$ i.e., random errors; $i=1(1)m$. The objective function to be minimized to obtain a fit is defined by *S*, the sum of squared residuals (observed $\boldsymbol{y_i}$ – expected $\boldsymbol{y_i}$). Subject to certain constraints, the estimated parameters then have minimum variance (from Gauss Markov Theorem). Also, from the theory of least squares, the following result [41] can be shown.

If *X* -be the matrix formed from the independent variables (form (X_{gj}); $g=1(1)k, j=1(1)n$) then the parameter estimate vector is given by formula (A4)

$$\hat{\beta} = (X^T X)^{-1} X^T Y, \tag{A4}$$

An unbiased estimate of σ^2 is given by $\frac{S}{(n-k)}$, and $\frac{S}{\sigma^2}$ follows a chi-square distribution with (n-k) degrees of freedom. Now, given the values X_{gj}, the corresponding value of the dependent variable may be calculated by formula (A5).

$$y_g = \sum_{j=1}^{n} X_{gj}\bar{\beta}_j, \tag{A5}$$

Further, representing the values of X_{gj} in vector form and denoting as M, the $100(1\text{-}\alpha)\%$ confidence interval for the response is given as follows by formula (A6).

$$M^T\hat{\beta} \pm t_{\frac{\alpha}{2},k-n}\hat{\sigma}\sqrt{1 + M^T(X^T X)^{-1}M}, \tag{A6}$$

where, "*t*" denotes the upper $(\alpha/2)^{th}$ point in *t*-distribution with (k-n) degrees of freedom. These intervals were constructed for each data set used for building the model.

Construction of Prediction Intervals: Consider a random sample of responses, X_1 , X_2 , … X_n. For such an interval an ancillary statistic [41] is required, which is one that completely depends on observables. Here the simplest: the sample mean, $\bar{X}$ and (unbiased) variance, s^2 as given below by formula (A7-A8) are utilised:

$$\bar{X} = \bar{X}_n = (X_1 + X_2 + \dots + X_n)/n, \tag{A7}$$

$$s^2 = s_n^2 = \sum_{i=1}^{n}(X_i - \bar{X}_n)^2/(n-1), \tag{A8}$$

Combining the above for a normal distribution $N(\mu,\sigma^2)$ with both μ and σ^2 unknown yields the following ancillary statistic:

$$(X_{n+1} - \bar{X}_n)/\left(s_n\sqrt{1+1/n}\right) \sim t_{n-1}, \tag{A9}$$

Here, X_{n+1} denotes the *(n+1)th* observation of the series to be predicted. This simple combination is possible because the sample mean and sample variance of the normal distribution are independent statistics. This is only true for the normal distribution, and in fact characterizes the normal distribution.

Solving further yields formula (A10) as the *100(1-α)%* prediction interval for X_{n+1},

$$\bar{X}_n \pm t_{\frac{\alpha}{2},n-1} s_n\sqrt{1+1/n}, \tag{A10}$$

where, "*t*" denotes the upper $(\alpha/2)^{th}$ point in *t*-distribution with (*n-1*) degrees of freedom.

Section E

In a least square approach it is important to understand the nature of the underlying residuals since one of the fundamental assumptions is that they are approximately normally distributed with mean as zero and a constant variance. Collinearity can be related to the existence of near-linear dependencies among the independent variables. Criteria such as Variance Inflation Factor and Breusch-Pagan test [40] have been used to explore the nature of the residuals and further to measure the collinearity imposed by different cofactors in the model.

Variance Inflation Factor (VIF): This provides an index to infer the increase in the variance of an estimated regression coefficient due to collinearity [40]. Let the independent variables in a multiple linear regression model be: $X_1, X_2, \ldots, X_k$ with regression coefficients, $\beta_1, \beta_2, \ldots, \beta_k$. Thus we consider the model from formula (A3) above where, ϵ_i 's are $N(0,\sigma^2)$ i.e., random errors; $i=1(1)m$. For each regressor, X_i , the *VIF* is given by formula (A11).

$$VIF_i = 1/(1 - R_i^2), \qquad \text{(A11)}$$

where R_i^2 is the coefficient of determination obtained in each of the auxillary regressions of the following form (formula (A12)):

$$X_i = \beta_0 + \sum_{\substack{j=1 \\ j \neq i}}^{n} \beta_j X_j + \epsilon_i' , \qquad i = 1(1)m, \qquad \text{(A12)}$$

Subsequently, R_i^2 are computed for each such model and the corresponding *VIFs* are obtained (formula (A11)). In case the *VIF* is greater than 5, then multi-collinearity is cited to be high

and points towards collinearity of a particular independent variable. In our case, the *VIFs* for the two initial models are as given in Table A7(a) and (b).

Table S7 (a) *Variance Inflation Factor for the initial model for SPR values of Chennai from January 2002 to December 2004*

**Independent variable**	**Statistic**	**Decision**
SPR_{-1}	1.2934	No *significant* collinearity
T	2.0716	No *significant* collinearity
T_{-1}	1.8135	No *significant* collinearity
P	4.2158	No *significant* collinearity
R	1.4967	No *significant* collinearity

SPR_{-1}: SPR-at-lag-one; T: Average Temperature; T_{-1}: Average Temperature-at-lag-one; P: Population; R: Rainfall;

Table S7 (b) *Variance Inflation Factor for the initial model for TMC values of Mangalore from January 2003 to December 2007*

**Independent variable**	**Statistic**	**Decision**
TMC_{-1}	1.9131	No *significant* collinearity
T	4.0209	No *significant* collinearity
T_{-1}	2.7855	No *significant* collinearity
R	2.172	No *significant* collinearity

TMC_{-1}:TMC-at-lag-one; T: Average Temperature;T_{-1}: Average Temperature-at-lag-one; R: Rainfall;

The values of VIFs in this case clearly indicate that there are no values beyond 5, which shows the absence of multi-collinearity (Table S7 (a) and (b)). Hence, it can concluded that

the chosen independent variables in both data, do not lead to violation of the assumption of constant variance of the residuals.

Breusch-Pagan Test: It is one of the commonly used statistical tests for heteroscedasticity diagnostics [40]. The model given by formula (A1) can be written as follows:

$$y_i = \overrightarrow{X_i}\vec{\beta} + \epsilon_i, \tag{A13}$$

where, $i=1(1)m$ and $\overrightarrow{X_i} = [1X_{2i}\, X_{3i} \ldots \ldots X_{ki}]$. We assume that heteroscedasticity takes the form:

$$E(\epsilon_i) = 0 \; for \; all \; i; \qquad \sigma_i^2 = E(\epsilon_i^2) = h(\overrightarrow{z_i}^T \vec{\alpha}); \tag{A14}$$

where, $\overrightarrow{z_i}^T = [1 \; z_{2i} z_{3i} \ldots z_{pi}]$ and $\vec{\alpha} = [\alpha_1 \alpha_2 \ldots \alpha_p]$ is a vector of unknown coefficients and $h(.)$ is an arbitrary function that must take only positive values. The null hypothesis of homoscedasticity is then:

$$H_o: \alpha_2 = \alpha_3 = \cdots = \alpha_p = 0, \tag{A15}$$

Under the null, we have $\sigma_i^2 = h(\alpha_1)$, that is, constant. Next, the coefficients are to be estimated from the original model equation using ordinary least squares. Let the square of the resulting residuals be $e_i^2, i = 1(1)m,$ which are then regressed on $\vec{z}^T$. This yields a coefficient of determination, R^2.

Finally the Breusch-Pagan statistic, mR^2, where m is the sample size, asymptotically follows a χ^2 distribution with $(p-1)$ degrees of freedom under the null hypothesis (formula (A16)).

$$mR^2 \sim \chi^2_{(p-1)}, \qquad (A16)$$

This test requires the knowledge of the regressors $\vec{z}^T$ but not that of the functional form *h(.)*.

The values of this statistic are given in the Table S8 for both the initial models. The homoscedastic null hypothesis is not rejected at 99% level of significance. This reinstates that the residuals show no erratic behavior as a function of the value of the responses. That is, it can be assumed that there is no significant level of heteroscedasticity. This provides a comprehensive reason for the use of our model fitting approach for the available data.

Table S8 *Breusch-Pagan Test results for verifying the null hypothesis of homoscedasticity for the initial models for SPR values for Chennai from January 2002 to December 2004 and TMC values of Mangalore from January 2003 to December 2007*

	**Chennai**	**Mangalore**
**Statistic**	7.219	12.6704
***p*-value**	0.2049	0.013
**Decision**	Not rejected at 99% level of significance	Not rejected at 99% level of significance
